# Manual ventilation quality is improved with a real-time visual feedback system during simulated resuscitation

**DOI:** 10.1186/s12245-020-00276-y

**Published:** 2020-04-16

**Authors:** Jeffrey R. Gould, Lisa Campana, Danielle Rabickow, Richard Raymond, Robert Partridge

**Affiliations:** 1 ZOLL Medical, Chelmsford, MA USA; 2 Armstrong Ambulance, Arlington, MA USA; 3 Department of Emergency Medicine, Emerson Hospital, 133 ORNAC, Concord, MA 01742 USA

**Keywords:** Airway, Cardiopulmonary resuscitation (CPR), Tidal volume, Ventilation

## Abstract

**Introduction:**

Manual ventilations during cardiac arrest are frequently performed outside of recommended guidelines. Real-time feedback has been shown to improve chest compression quality, but the use of feedback to guide ventilation volume and rate has not been studied. The purpose of this study was to determine whether the use of a real-time visual feedback system for ventilation volume and rate improves manual ventilation quality during simulated cardiac arrest.

**Methods:**

Teams of 2 emergency medical technicians (EMTs) performed two 8-min rounds of cardiopulmonary resuscitation (CPR) on a manikin during a simulated cardiac arrest scenario with one EMT performing ventilations while the other performed compressions. The EMTs switched roles every 2 min. During the first round of CPR, ventilation and chest compression feedback was disabled on a monitor/defibrillator. Following a 20-min rest period and a brief session to familiarize the EMTs with the feedback technology, the trial was repeated with feedback enabled. The primary outcome variables for the study were ventilations and chest compressions within target. Ventilation rate (target, 8–10 breaths/minute) and tidal volume (target, 425–575 ml) were measured using a novel differential pressure-based flow sensor. Data were analyzed using paired *t* tests.

**Results:**

Ten teams of 2 EMTs completed the study. Mean percentages of ventilations performed in target for rate (41% vs. 71%, *p* < 0.01), for volume (31% vs. 79%, *p* < 0.01), and for rate and volume together (10% vs. 63%, *p* < 0.01) were significantly greater with feedback.

**Conclusion:**

The use of a novel visual feedback system for ventilation quality increased the percentage of ventilations in target for rate and volume during simulated CPR. Real-time feedback to perform ventilations within recommended guidelines during cardiac arrest should be further investigated in human resuscitation.

## Introduction

High-quality ventilations are a critical component of cardiac arrest resuscitation; however, it is known that ventilations are commonly performed outside of recommended guidelines [1, 2]. Hyperventilation is associated with adverse hemodynamic effects [3, 4]; higher tidal volumes and end-expiratory pressures increase pulmonary vascular resistance and reduce cardiac output [5]. Currently, there are no reliable tools available that provide real-time ventilation feedback to guide rescuers during resuscitation.

Properly delivered manual ventilations are challenging to achieve during resuscitation. During cardiac arrest, oxygen delivery to critical organs is limited by blood flow rather than arterial oxygen content [6, 7]. A ventilation bag may be used to perform rescue breaths before and after placement of an advanced airway, unless a mechanical ventilator is used. During any type of manual ventilation, adequate tidal volumes can be difficult to achieve, and complications are well described [8]. It is known that healthcare professionals commonly perform ventilations outside of recommended guidelines during CPR [3, 9] and that higher respiratory rates and elevated tidal volumes are associated with poor outcomes.

**Fig. 1:**
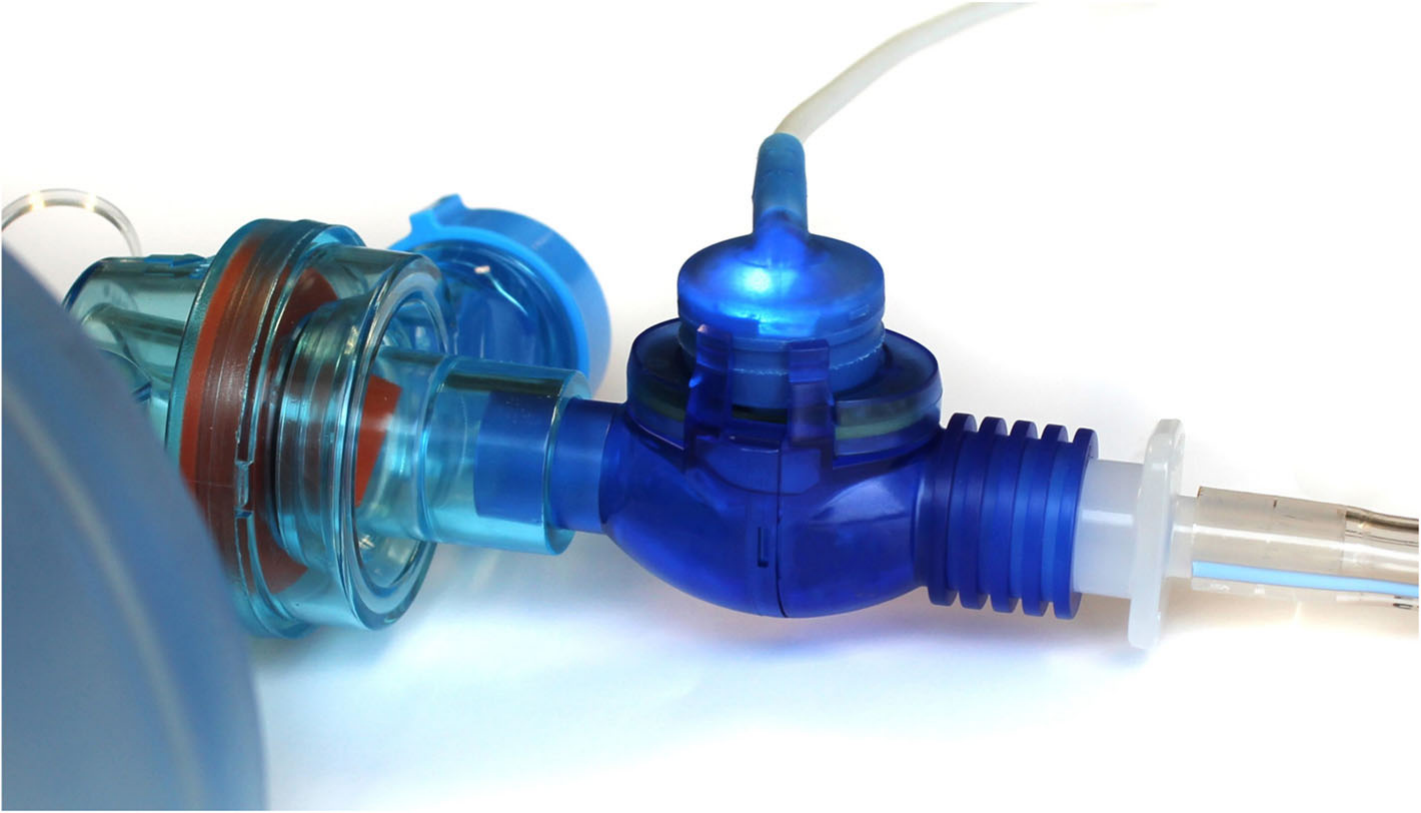
Disposable differential pressure-based flow sensor used to measure ventilation rate and tidal volume. Shown attached to a ventilation bag, endotracheal tube, and a reusable cable (ZOLL Medical, AccuVent)

**Fig. 2:**
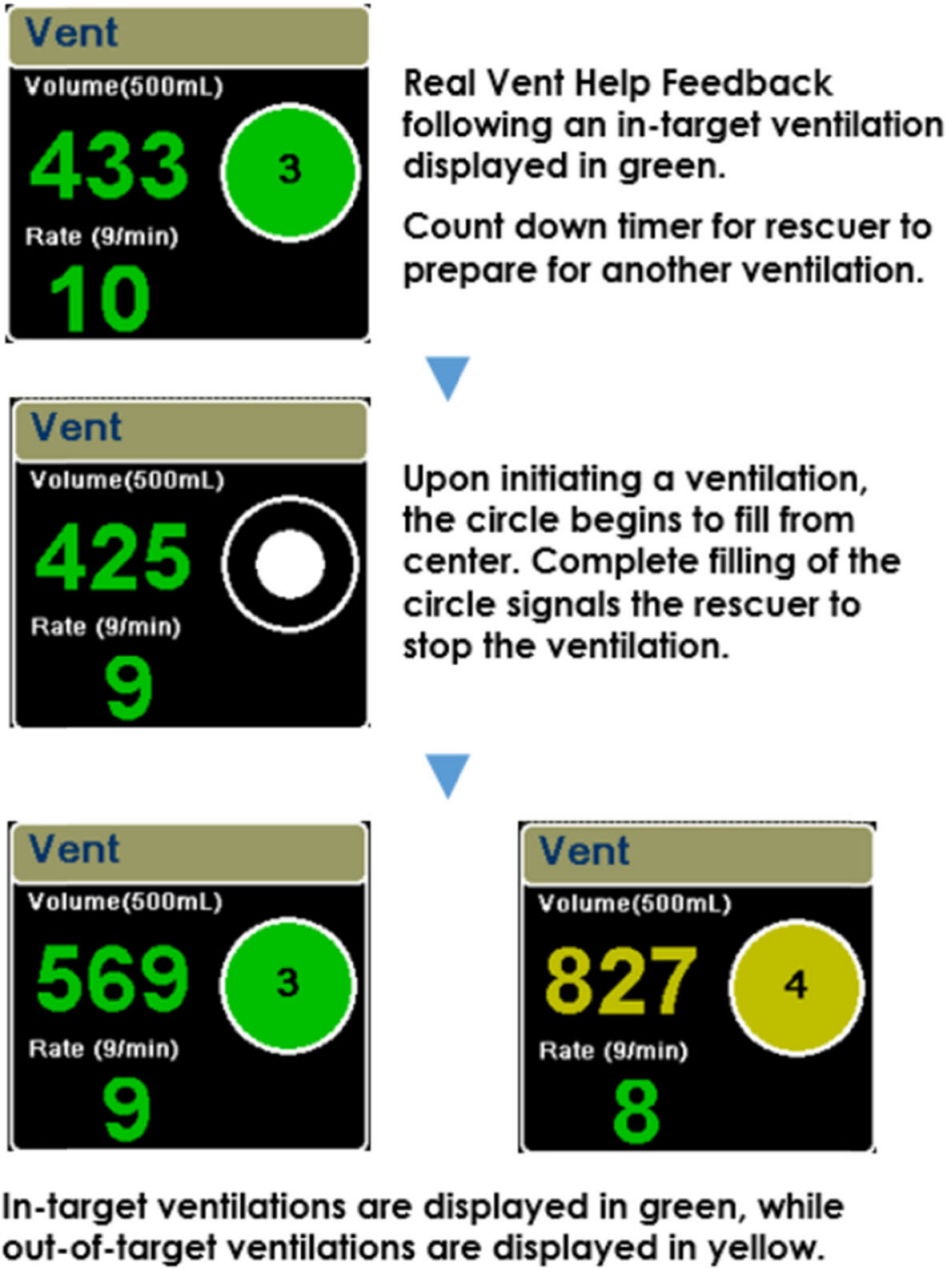
Real Vent Help dashboard display of real-time feedback on ZOLL X series defibrillator/monitor

A new technology (AccuVent™, ZOLL Medical, Chelmsford, MA) has been developed to provide real-time feedback on ventilation quality to healthcare professionals during resuscitation. This technology comprises a differential pressure-based flow sensor that is placed between the ventilation bag and airway to measure respiratory rate and ventilation volume during resuscitation. This information is then displayed numerically and graphically on a defibrillator/monitor in real time to guide rescuers in delivering manual ventilations within recommended guidelines (Fig. 1, Fig. 2).

Real-time feedback has previously been shown to improve chest compression quality and patient survival during human resuscitation [10–12]. However, the effects of real-time feedback to guide delivery of ventilations at the appropriate rate and tidal volume have not been examined. The purpose of this study was to determine whether the use of a real-time visual feedback system improves the quality of manual ventilations during simulated cardiac arrest.

## Methods

Basic life support (BLS) and advanced life support (ALS) emergency medical technicians (EMTs) volunteered for the study and worked in pairs. Potential participants were excluded if they were CPR instructors, had known health problems that could limit physical performance, or were pregnant. In an effort to minimize the Hawthorne effect, the true purpose of the study was not disclosed to participants. Instead, they were told that the study was investigating levels of fatigue experienced while performing CPR.

This investigation was approved by the New England Institutional Review Board (IRB #: 120170151). Written informed consent was obtained from all participants. For each pair of participants, the study involved a 1-hour session, which included being familiarized with the study protocol, completing a demographic questionnaire, and performing two simulated cardiac arrest trials.

### Simulated cardiac arrest trials

Participants worked in pairs to perform two trials of a simulated cardiac resuscitation on an intubated manikin (Simulaids, Saugerties, NY). A monitor/defibrillator (X Series®, ZOLL Medical, Chelmsford, MA) was placed next to the manikin and training defibrillator pads with an accelerometer-based sensor were placed on the chest. During each trial, EMTs performed CPR for a total of 8 min. One EMT performed compressions while the other performed manual ventilations using a ventilation bag. EMTs switched roles every 2 min. Each EMT performed 2 rounds of compressions and 2 rounds of ventilations during each trial.

During the first trial, ventilation and chest compression feedback features were disabled on the defibrillator/monitor. Following a 20-min rest period and a brief session to familiarize participants with the feedback dashboards displayed on the monitor, the trial was repeated with real-time feedback on ventilation and compression quality enabled on the monitor.

### Outcome variables

The primary outcome variables for the study were ventilations and chest compressions within target, measured before and after the intervention of real-time feedback. Ventilation rate (target, 8–10 breaths/minute) and tidal volume (target, 500 ± 75 ml) were measured using the AccuVent™ sensor. Real-time feedback showing rate and volume of each ventilation was displayed numerically and graphically on the monitor (Fig. 2). Chest compression depth (target, 2–2.5 in.) and rate (target, 100–120 compressions/minute) were measured using an accelerometer-based system embedded within the electrodes placed on the manikin’s chest (Real CPR Help, ZOLL).

Paired *t* tests were used to compare trials performed without feedback to those from the second trial with feedback enabled.

## Results

Twenty EMTs (4 females) with a range of clinical experience participated in the study. The median age of participants was 28 years (IQR = 23–40 years). All participants held current CPR certification, 13 held basic life support (BLS) certification, and 7 held advanced cardiac life support (ACLS) certification. The median time spent employed in emergency medical services (EMS) was 3 years (IQR = 2–17 years) (Table 1).

**Table 1:** Participant characteristics

*n*	20 (4 females)
Age (years)	28 (IQR 23,40)
Years employed in EMS	3 (IQR 2,17)
Training (*n*)	
BLS certified	13
ACLS certified	7
Last certification (*n*)	
< 3 months	9
3–6 months	1
6–12 months	6
> 12 months	4
Number of times performing compressions during human cardiac arrest (*n*)	
Never	4
1–5	3
6–10	5
11–15	0
16–20	0
> 20	8
Number of times performing ventilations during human cardiac arrest (*n*)	
Never	2
1–5	4
6–10	1
11–15	3
16–20	1
> 20	9

Age and years employed in EMS presented as median (IQR)

Mean percentages of ventilations performed in target for rate (41% vs. 71%, *p* < 0.01), for volume (31% vs. 79%, *p* < 0.01), and for rate and volume together (10% vs. 63%, *p* < 0.01) were significantly greater with feedback. Similarly, the percentage of chest compressions that were performed in target for rate (36% vs. 76%, *p* < 0.01), for depth (34% vs. 70%, *p* < 0.01), and for rate and depth together (16% vs. 55%, *p* < 0.01) was significantly greater with the use of visual feedback (Table 2).

**Table 2:** Ventilations and compressions performed in target with and without the use of real-time feedback

	No feedback	Feedback	*P*
Compressions in target for rate (%)	36 ± 36	76 ± 26	< 0.01
Compressions in target for depth (%)	34 ± 30	70 ± 25	< 0.01
Compressions in target for rate and depth (%)	16 ± 25	55 ± 28	< 0.01
Ventilations in target for rate (%)	41 ± 23	71 ± 16	< 0.01
Ventilations in target for volume (%)	31 ± 32	79 ± 15	< 0.01
Ventilations in target for rate and volume (%)	10 ± 14	63 ± 18	< 0.01

Data presented as mean ± standard deviation

## Discussion

This investigation demonstrated that real-time ventilation feedback, used in simulated cardiac arrest resuscitation, improves ventilation quality. In addition, simultaneous display of real-time compression feedback improved chest compression quality, demonstrating that the improvement in ventilation quality did not come at the expense of compression quality. Prior studies have shown that real-time feedback improves compression quality [10], and improved compression quality has been shown to improve patient survival [11, 12]. Real-time feedback for ventilation rate and volume has not previously been reported.

Ventilations performed by health care professionals during resuscitations are frequently performed outside of recommended guidelines for both rate and tidal volume [2, 3, 9]. Excessive ventilation rate and/or tidal volume have been shown to be detrimental to survival [1]. Providing real-time feedback on ventilation rate and tidal volume to rescuers may improve rescuer performance according to established resuscitation guidelines and as a result may reduce the risk of detrimental outcome due to excessive ventilation rates and tidal volumes. Avoiding hyperventilation and excessive tidal volumes may result in improved patient survival.

## Limitations

The main limitation of the study is that the order of the CPR trials was not randomized, although this design has been successfully used in other feedback studies. All participants performed CPR without feedback in the first trial. While it is possible that a learning effect may have led to an increase in compression and ventilation quality in the second trial, a secondary analysis showed that the number of compressions and ventilations in target during the first 2-min round did not differ from the second 2-min round. A lack of improvement within trials suggests that any potential learning effect was minimal.

Interpretation of the findings in this study can only be applied to the simulation setting. Although the use of ventilation feedback in patient resuscitation is feasible, other factors associated with actual resuscitation may adversely affect the performance of the ventilation monitoring technology. Human studies using real-time ventilation feedback during resuscitation will be useful to determine the effectiveness of ventilation feedback technology in the clinical setting and determine whether real-time feedback on ventilation quality improves outcome after cardiac arrest.

## Conclusion

The use of a ventilation feedback system in simulated cardiopulmonary resuscitation improved ventilation quality by increasing the number of manual ventilations performed within target for rate and volume. This technology may address an unmet need in resuscitation medicine. Real-time ventilation feedback during resuscitation should be further studied in humans to determine the effectiveness of feedback technology on ventilation quality and outcome after cardiac arrest.

## Data Availability

The datasets used and/or analyzed during the current study are available from the corresponding author on reasonable request and with permission of Zoll Medical Corporation.

## Abbreviations

EMTs: Emergency medical technicians
CPR: Cardiopulmonary resuscitation
BLS: Basic life support
ALS: Advanced life support
IRB: Institutional Review Board
ACLS: Advanced cardiac life support
EMS: Emergency medical services
